# Incidence of microvascular complications of type 2 diabetes: A 12 year longitudinal study from Karachi-Pakistan

**DOI:** 10.12669/pjms.345.15224

**Published:** 2018

**Authors:** Asher Fawwad, Nida Mustafa, Awn Bin Zafar, Maria Khalid

**Affiliations:** 1 Asher Fawwad, PhD. Associate Professor, Department of Biochemistry Senior Research Scientist, Department of Research, Baqai Institute of Diabetology and Endocrinology, Baqai Medical University, Karachi, Pakistan; 2 Nida Mustafa, M.Sc. Statistician, Department of Research, Baqai Institute of Diabetology and Endocrinology, Baqai Medical University, Karachi, Pakistan; 3 Awn Bin Zafar, M.C.P.S. Assistant Professor, Department of Medicine, Baqai Institute of Diabetology and Endocrinology, Baqai Medical University, Karachi, Pakistan; 4 Maria Khalid, M.B.B.S. Research Officer, Department of Research, Baqai Institute of Diabetology and Endocrinology, Baqai Medical University, Karachi, Pakistan

**Keywords:** Incidence rate, Microvascular complications, Type 2 diabetes

## Abstract

**Objectives:**

To estimate the incidence of microvascular complications among subjects with type 2 diabetes at a tertiary care hospital.

**Methods:**

This retrospective longitudinal follow-up study assessed the data records of type 2 diabetic subjects who visited the outpatient department of Baqai Institute of Diabetology and Endocrinology, Baqai Medical University, from January 2005 to April 2016. Subjects with gestational diabetes, type 1 diabetes and with history of any microvascular complication were excluded. Medical records were obtained through electronic database (Health Management System). Statistical analyses were conducted using STATA version 14 and SPSS version 20.

**Results:**

The incidence of microvascular complications was 92.8, 106.2, and 130.2 per 1000 person per years for retinopathy, neuropathy and nephropathy respectively. Retinopathy, neuropathy and nephropathy were significantly high among diabetic patients with duration of diabetes >10 years followed by 5-10 years. Incidence of retinopathy and nephropathy was significantly higher in patients who had HbA1c>7% than patients with HbA1c≤7% (p-value<0.05). Higher incidence rate of all three microvascular complications were seen in subjects with hypertension than subjects without hypertension.

**Conclusion:**

A high incidence of microvascular complications is found in subjects with type 2 diabetes. Poor glycaemic control, longer duration of diabetes and hypertension was found to be associated with the occurrence of these complications.

## INTRODUCTION

Diabetes mellitus is a global pandemic for the 21^st^ century.^1^ Subjects with type 2 diabetes can have metabolic and vascular complications.^2^ Chronic complications can be classified as macrovascular or microvascular complications.^1^ Epidemiologic study has shown a fifth of all diabetes patients suffer from two or more micro vascular complications. The complications give rise to morbidity and compromise the quality of life for diabetic subjects.^3^ Long-term complications of diabetes include retinopathy with potential loss of vision; nephropathy leading to renal failure; peripheral neuropathy with risk of foot ulcers, amputations, and Charcot joints; and autonomic neuropathy causing gastrointestinal, genitourinary, and cardiovascular symptoms and sexual dysfunction.^4^ There are a number of internationally recognized guidelines, algorithms, and position statements for the diagnosis, control, and management of diabetes. However, implementation of guidelines for the management of diabetes has beneficial effects for the individual with diabetes, including a significant decrease in complications associated with type 2 diabetes.^5^

The American Diabetes Association (ADA) has designated HbA1C level of <7% as a target to control blood glucose^6^ and International Diabetes Federation (IDF) has designated HbA1c of <6.5 for optimal blood glucose control.^7^ According to United Kingdom Prospective Diabetes Study (UKPDS) and Kumamoto study, risk of microvascular complications can be lowered after achieving adequate glycemic control. Similarly, a hypertensive subgroup analyzed in the UKPDS showed adequate control of blood pressure improves the outcome of both macrovascular and microvascular complications.^8^,^9^ At the age of 45, around 40% of subjects with type 2 diabetes are hypertensive, the proportion increasing to 60% by the age of 75.^9^ With the holistic approach, these complications can be controlled leading to reduction in morbidity, mortality and healthcare costs.^2^ The present study, therefore, aims to estimate the incidence of chronic microvascular complications in a large sample of type 2 diabetic subjects attending a tertiary care hospital.

## METHODS

This retrospective longitudinal follow-up study assessed the data records of type 2 diabetic subjects visiting the outpatient department of Baqai Institute of Diabetology and Endocrinology (BIDE), Baqai Medical University, Karachi - Pakistan from January 2005 to April 2016. Subjects with gestational diabetes, type 1 diabetes and a history of any microvascular complications at first visit, were excluded. Medical records were obtained through electronic database (Health Management System). The records of subjects, included as per the inclusion criteria having no complication at baseline visit were followed for their follow up visits to document the development of any microvascular complication.

Retinopathy was labelled on finding the diagnostic signs of retinopathy on eye exam i.e., development of pre-proliferative & proliferative retinopathy, macular edema or diabetes-related blindness by direct fundoscopy. Neuropathy was diagnosed as per the standardized guidelines after checking pin prick, vibration sense, ankle reflex and knee reflex. Whereas, diagnosis of nephropathy was based on the findings of urinary albumin in urine detailed report, urinary *micro albumin* and 24 hours’ urine for protein and creatinine clearance.^2^,^8^ Blood pressure was measured by mercury sphygmomanometer in a sitting position by using standard method. Average blood pressure over the follow-up period was determined from the blood pressure recorded at each visit. Average blood pressure level≤130/85 mmHg was taken as normal or controlled BP and average blood pressure level >130/85 mmHg was taken as high BP.^10^ AverageHbA1c value of ≤7% and >7% were taken as good and poor control respectively.^2^,^8^ Average HbA1c was defined as the mean of all HbA1c values from baseline until the development of any microvascular complication or censoring. HbA1c was performed by HPLC method on BIO RAD D-10.

### Statistical Analysis

The incidence of microvascular complications in the follow-up period was estimated by number of observed new microvascular complications cases per 1000 person per years. Follow up time (person-year) was calculated as the time elapsed from the date of registration till the date of microvascular complication development, or the end of follow-up, whichever came first. The calculation of 95% confidence interval for the incidence rate was based on the assumption that the observed incidence cases followed a Poisson distribution. Incidence were estimated by different intervals of duration of diabetes, glycemic control and blood pressure control. T-test and chi-square test were also applied to determine the significance. A two-sided P-value<0.05 was considered statistically significant. Statistical analyses were conducted using STATA version 14 and SPSS version 20.

### Ethical approval

Study was approved from the Institutional Review Board of Baqai Institute of Diabetology and Endocrinology, Baqai Medical University.

## RESULTS

A total of 4633 type 2 diabetic subjects were included in this study. Among them 2336 (50.4%) were male and 2297(49.6%) were female. Large number of subjects (55.7%) had duration of diabetes >5 years. At baseline, 3175 (85.9%) of the subjects had HbA1c level >7% and 1976(57.2%) were hypertensive (Table-I).

**Table-I:** Basic and Clinical Characteristics.

Variables	Female	Male	P-value	Overall
N	2297	2336	-	4633
Age (years)	50.7±10.26	50.68±11.22	0.938	50.69±10.76
BMI (kg/m^2^)	29.28±5.58	27.35±4.75	<0.0001	28.29±5.26
***Duration of diabetes***				
<5 years	968(42.1%)	1086(46.5%)	0.011	2054(44.3%)
5-10 years	700(30.5%)	647(27.7%)		1347(29.1%)
>10 years	629(27.4%)	603(25.8%)	1232(26.6%)
***Family history of diabetes***				
Yes	1210(71.1%)	1209(67.7%)	0.028	2419(69.4%)
No	491(28.9%)	577(32.3%)	1068(30.6%)
***Smoking history***				
Current smoker	6(0.4%)	302(17%)	<0.0001	308(8.9%)
Never Smoked	1682(99.6%)	1477(83.0%)	3159(91.1%)
***Alcohol history***				
Current drinker	1(0.1%)	18(1.0%)	<0.0001	19(0.6%)
Never drink	1682(99.9%)	1746(99.0%)	3428(99.4%)
***Hypertension status***				
Yes	1047(62.2%)	929(52.4%)	<0.0001	1976(57.2%)
No	635(37.8%)	844(47.6%)	1479(42.8%)
***HbA1c (%)***				
≤7%	237(13.2%)	283(14.9%)	0.139	520(14.1%)
>7%	1558(86.8%)	1617(85.1%)		3175(85.9%)
Cholesterol (mg/dL)	183.36±43.76	175±45.08	<0.0001	178.96±44.65
HDL (mg/dL)	40.11±10.15	36.65±8.29	<0.0001	38.29±9.38
LDL (mg/dL)	111.83±35.05	106.83±33.06	<0.0001	109.2±34.1
Creatinine (mg/dL)	0.94±0.43	1.1±0.36	<0.0001	1.02±0.41
Triglyceride (mg/dL)	176.26±127.76	181.22±139.73	0.348	178.88±134.21

Data presented as mean±SD or n (%) P-value <0.05 was considered statistically significant.

The incidence of microvascular complications was 92.8, 106.2, and 130.2 per 1000 person per years for retinopathy, neuropathy and nephropathy respectively. The incidence rate ratio (IRR) shows that retinopathy, neuropathy, and nephropathy were significantly higher among diabetic patients with duration of diabetes >10 years followed by 5-10 years (Table-II).

**Table-II:** Incidence of Microvascular complications according to duration of diabetes, HbA1c level, Hypertension status.

	Follow up time (personyear)	Development of Microvascular complication	Incidence density (1000 personyear)	IRR (95% C.I)	P-value
***Duration of Diabetes***					
***Retinopathy***					
<5 years	1226.997	49	39.93	1	
5-10 years	573.48	60	104.62	2.61(1.76-3.90)	<0.0001
>10years	578.784	112	193.5	4.84(3.43-6.92)	<0.0001
***Neuropathy***					
<5 years	3390.25	360	106.18	1	
5-10 years	1682.23	239	142.07	1.33(1.13-1.58)	0.0006
>10years	1065.41	200	187.72	1.76(1.47-2.10)	<0.0001
***Nephropathy***					
<5 years	4927.66	446	90.5	1	
5-10 years	3251.65	357	109.79	1.21(1.05-1.39)	0.0068
>10years	2589.98	341	131.66	1.45(1.25-1.67)	<0.0001
***Average HbA1c***					
***Retinopathy***					
≤7%	262.424	9	34.29	1	0.0003
>7%	2102.134	209	99.42	2.89 (1.498 -6.428)
***Neuropathy***					
≤7%	763.08	90	117.94	1	0.328
>7%	5289.01	696	131.59	1.11 (0.894-1.405)
***Nephropathy***					
≤7%	1131.62	81	71.57	1	0.0001
>7%	9535.22	1047	109.8	1.53 (1.222-1.947)
***Average blood pressure***					
***Retinopathy***					
≤130/85 mmHg	1356.099	105	77.42	1	0.0047
>130/85 mmHg	1023.162	116	113.37	1.46 (1.114-1.925)
***Neuropathy***					
≤130/85 mmHg	3625.47	419	105.01	1	0.0002
>130/85 mmHg	2512.89	380	136.78	1.30 (1.136-1.507)
***Nephropathy***					
≤130/85 mmHg	5915.27	552	93.31	1	<0.0001
>130/85 mmHg	4820.44	586	121.56	1.30 (1.157-1.465)	

P-value <0.05 was considered statistically significant, IRR: incidence rate ratio.

Incidence of retinopathy and nephropathy was significantly higher in patients who had HbA1c>7% (IRR of retinopathy= 2.89; IRR of nephropathy =1.53, p-value<0.001) than patients with HbA1c≤7%. Similar trend was observed in neuropathy however, the difference was not significant (Table-II).

Furthermore, it was identified that incidence of all microvascular complications i.e. retinopathy, nephropathy and neuropathy was significantly higher among diabetics with hypertension (IRR of retinopathy=1.46; IRR of neuropathy=1.30; IRR of nephropathy= 1.30, p-value<0.005) than diabetics without hypertension (Table-II).

## DISCUSSION

The rising incidence of diabetes related complications poses a major clinical, societal and economic burden not only in high-income countries but also in developing countries.^11^ Various epidemiological studies have reported the role of several risk factors for progression of diabetes complications.^12^-^18^ However, there is a paucity of data regarding incidence of diabetes complication while the role of different risk factors for developing these complications are somewhat well understood in developing countries like Pakistan.^2^,^8^,^10^ This retrospective cohort study revealed the incidence of diabetes related micro vascular complications in subjects attending a tertiary care unit in Karachi, Pakistan. This study reported incidence rate of 92.9, 130.2 and 106.2 per 1000 person per years for retinopathy, neuropathy and nephropathy respectively. These figures are high as compared to the findings of other regional and international studies.^19^-^22^ The reason for high incidence is that the current study is from a tertiary care unit having more subjects with longer duration of diabetes. Further larger scale prospective and epidemiological studies are required to ascertain the findings of this study.

It is well documented in previous studies of India, Bangladesh, UAE, Egypt, and England that microvascular complications advance with duration of diabetes.^14^-^18^ The current study provided consistent outcomes of higher incidence of all three microvascular complications with the increasing duration of diabetes. Similar results were also observed in our previous study reporting high prevalence of microvascular complications with the advancement of duration of type 2 diabetes.^2^

Poor glycemic control has been considered as a major risk factor for microvascular complications. In this study, the incidence of microvascular complications (retinopathy and nephropathy) were significantly higher among type 2 diabetic subjects with uncontrolled HbA1c (HbA1c > 7%). Likewise, studies from Japan and Taiwan found significantly increased incidence of microvascular complications with higher levels of HbA1c.^19^,^23^ The trend for the development of neuropathy and incidence although shows the similar finding but it does not reach the level of significance. Authors from Lahore, Pakistan reported the same phenomenon that neuropathy was not significantly prevalent in subjects with uncontrolled diabetes.^6^ Using more advanced and sensitive gadgetries might improve the screening and thus their relationship with diabetes control can be ascertained in an improved manner.^24^

Control of blood pressure is another important hallmark in the management of type 2 diabetes to improve the mortality and morbidity.^25^ We observed strong and significant association of blood pressure control with retinopathy, neuropathy and nephropathy. Other regional and international studies are also in agreement with our finding.^12^,^13^,^21^,^26^

Strengths and limitations of the present study included its large sample size with 12 years follow-up to assess the incidence and occurrence of microvascular complications, probably first time from Pakistan. In the absence of community based epidemiological studies this data can be considered as a representative but future prospective and epidemiological studies are required to ascertain the findings of this study.

## CONCLUSION

*A high incidence of microvascular complications is found in subjects with type 2 diabetes. Poor glycaemic control, longer duration of diabetes and hypertension was found to be associated with the occurrence of these complications*.

### Authors’ Contributions

**FA:** Concept and design, undertook the data analyses, edited and revised the manuscript.

**MN:** Interpretation of data, prepared and reviewed the manuscript.

**ZAB:** Edited and revised the manuscript.

**KM:** Prepared and reviewed the manuscript.
